# Effects of a Digital Patient Empowerment and Communication Tool on Metabolic Control in People With Type 2 Diabetes: The DeMpower Multicenter Ambispective Study

**DOI:** 10.2196/40377

**Published:** 2022-10-03

**Authors:** Domingo Orozco-Beltrán, Cristóbal Morales, Sara Artola-Menéndez, Carlos Brotons, Sara Carrascosa, Cintia González, Óscar Baro, Alberto Aliaga, Karine Ferreira de Campos, María Villarejo, Carlos Hurtado, Carolina Álvarez-Ortega, Antón Gómez-García, Marta Cedenilla, Gonzalo Fernández

**Affiliations:** 1 Department of Clinical Medicine Miguel Hernandez University Alicante Spain; 2 Department of Endocrinology Hospital Universitario Virgen Macarena Seville Spain; 3 Department of Endocrinology Hospital Vithas Seville Spain; 4 José Marvá Heath Care Center RedGDPs Foundation Madrid Spain; 5 Sardenya Primary Health Care Center Biomedical Research Institute Sant Pau Barcelona Spain; 6 El Campello Primary Health Care Center Alicante Spain; 7 Department of Endocrinology and Nutrition Hospital de la Santa Creu i Sant Pau Centro de Investigacion Biomèdica En Red- Biotecnologia Biomedicina Nanotecnologia Barcelona Spain; 8 Galapagar Primary Care Center RedGDPs Foundation Madrid Spain; 9 Department of Endocrinology Clínica New Technologies in Diabetes and Endocrinology Seville Spain; 10 Medical Affairs Department MSD Spain Madrid Spain

**Keywords:** empowerment, home digital tool, telemedicine, type 2 diabetes, diabetic, home based, home care, self-management, digital tool, metabolic, HbA, glycated hemoglobin, glycemic control, adherence, satisfaction, observational study, health app

## Abstract

**Background:**

Diabetes is a major health care problem, reaching epidemic numbers worldwide. Reducing hemoglobin A_1c_ (HbA_1c_) levels to recommended targets is associated with a marked decrease in the risk of type 2 diabetes mellitus (T2DM)–related complications. The implementation of new technologies, particularly telemedicine, may be helpful to facilitate self-care and empower people with T2DM, leading to improved metabolic control of the disease.

**Objective:**

This study aimed to analyze the effect of a home digital patient empowerment and communication tool (DeMpower App) on metabolic control in people with inadequately controlled T2DM.

**Methods:**

The DeMpower study was multicenter with a retrospective (observational: 52 weeks of follow-up) and prospective (interventional: 52 weeks of follow-up) design that included people with T2DM, aged ≥18 and ≤80 years, with HbA_1c_ levels ≥7.5% to ≤9.5%, receiving treatment with noninsulin antihyperglycemic agents, and able to use a smartphone app. Individuals were randomly assigned (2:1) to the DeMpower app–empowered group or control group. We describe the effect of empowerment on the proportion of patients achieving the study glycemic target, defined as HbA_1c_≤7.5% with a ≥0.5% reduction in HbA_1c_ at week 24.

**Results:**

Due to the COVID-19 pandemic, the study was stopped prematurely, and 50 patients (33 in the DeMpower app–empowered group and 17 in the control group) were analyzed. There was a trend toward a higher proportion of patients achieving the study glycemic target (46% vs 18%; *P*=.07) in the DeMpower app group that was statistically significant when the target was HbA_1c_≤7.5% (64% vs 24%; *P*=.02) or HbA_1c_≤8% (85% vs 53%; *P*=.02). The mean HbA_1c_ was significantly reduced at week 24 (−0.81, SD 0.89 vs −0.15, SD 1.03; *P*=.03); trends for improvement in other cardiovascular risk factors, medication adherence, and satisfaction were observed.

**Conclusions:**

The results suggest that patient empowerment through home digital tools has a potential effect on metabolic control, which might be even more relevant during the COVID-19 pandemic and in a digital health scenario.

## Introduction

Diabetes is a major health care problem, reaching epidemic numbers worldwide [1,2]. Globally, approximately 537 million people had type 2 diabetes mellitus (T2DM) in 2021, but it is expected that these numbers will increase up to nearly 783 million people by 2045 due to aging populations and the negative impact of some lifestyles, such as obesity and sedentarism [1-3]. This translates into a huge socioeconomic impact in addition to the health care burden [4]. In Spain, the prevalence of T2DM is estimated to be approximately 14% [5], and the direct health costs of diabetes account for approximately 8% of total public health expenditures [6].

Reducing hemoglobin A_1c_ (HbA_1c_) levels to recommended targets is associated with a marked decrease in the risk of T2DM-related complications [7]. Although adopting a healthy lifestyle (diet and physical activity) is necessary in T2DM to improve metabolic control, most people with T2DM will need at least 1 antidiabetic agent to control blood glucose levels. The pharmacological options to treat hyperglycemia in T2DM have improved substantially over the past 20 years with the development of new therapeutic agents that not only safely reduce HbA_1c_ levels but also have cardiovascular and renal benefits; unfortunately, many people with T2DM do not achieve recommended HbA_1c_ targets (<7%) [8,9]. In Spain, the proportion of people with T2DM with good glycemic control has not improved markedly over the last decade, remaining at around 50%-60%, suggesting that additional approaches are warranted [10-12]. Moreover, the lockdown during the COVID-19 pandemic has led to a worsening of follow-up and metabolic control in people with T2DM globally and in Spain [13-16].

Although many causes have emerged to explain this poor metabolic control in people with T2DM, poor adherence to treatment and clinical inertia play a key role [17-19]. Therefore, proper management of T2DM is challenging and deserves constant attention and comprehensive patient-centered clinical assistance. Consequently, it is necessary to transform health care systems to provide integrated and patient-centered chronic care models [20].

In this context, the implementation of new technologies, particularly telemedicine, may be helpful to facilitate patient self-care and empowerment [21,22]. In fact, effective diabetes self-management is a key goal, but it should be measured and monitored as part of routine care and technology may help patients and guide clinical decisions [22]. Different studies have shown that the use of telemedicine is associated with improvements in patients’ outcomes such as adherence, pathology control, and engagement [21,23-27]. However, in Spain, there are few studies evaluating eHealth solutions for people with T2DM, mostly developed in small local settings [28-33].

Taking into account the high prevalence and burden of T2DM in Spain and the current high number of people with inadequate metabolic control, developing innovative solutions to improve this situation is necessary. This improvement should be made through patient empowerment by increasing self-management and communication between patients and health care professionals, allowing more effective T2DM control.

The aim of this study was to analyze the effect of a home digital patient empowerment and communication tool (DeMpower App) on metabolic control in people with T2DM and inadequate HbA_1c_ levels compared to a control group, both treated according to usual clinical practice.

## Methods

### Overview

The DeMpower study was a multicenter and an ambispective study including adults with T2DM having inadequate glycemic control, treated according to clinical practice across Spain. The study population included people with T2DM aged ≥18 and ≤80 years from Spanish health care sites with HbA_1c_ levels ≥7.5% and ≤9.5%, who were receiving treatment with noninsulin antihyperglycemic agents and who were able to use a smartphone-based home digital tool. The main exclusion criteria were the use of insulin treatments, pregnancy, any scheduled surgery, terminal or severe diseases, or any medical or psychological condition that, in the investigator’s opinion, might have compromised the ability of the patient to provide informed consent. Patients were recruited consecutively as they visited the doctor’s office, reducing the possibility of selection bias and strengthening the generalizability of the results.

The enrollment period was approximately 12 months and patients were followed up for 52 weeks. The primary end point was assessed at week 24 of follow-up. Retrospective data were collected during the 52 weeks prior to the baseline visit, and the HbA_1c_ determination closest to the 24 weeks before baseline and the antidiabetic treatment prescribed at that time were recorded. After the enrollment period, patients were randomly assigned (2:1) to two comparative groups: group 1 (DeMpower app–empowered group), where patients were clinically managed according to usual clinical practice and used the DeMpower app during the prospective study follow-up, and group 2 (control group), where patients were clinically managed according to usual clinical practice without the DeMpower app. After the primary assessment at week 24, patients in group 1 were randomized again (1:1) to assess the durability of the effect at week 52: group 1a (DeMpower app–empowered group, long-term use), where patients kept using the DeMpower app, and group 1b (DeMpower app–empowered group, short-term use), where patients stopped using the DeMpower app. Both groups continued being clinically managed according to usual clinical practice (Figure S1 in Multimedia Appendix 1). The follow-up of group 2 continued without changes.

In group 1, patients received the following commercially available devices to use in combination with and connected to the DeMpower app: scale, glucometer, blood pressure monitor, and activity wristband. Patients were also trained to use the devices according to routine clinical practice, as agreed with their health care professionals (ie, taking periodic measurements of their glucose and blood pressure levels as well as their weight and degree of physical activity). Data from these devices (body weight, glucose levels, blood pressure, and number of steps taken daily) were received wirelessly by the DeMpower app for each patient and sent to the corresponding health care team to review the patient’s activity and measurements, answer patient questions, and contact the patient, when needed (Figure S2 in Multimedia Appendix 1). However, this channel of direct communication did not substitute clinical practice, and if health care was required due to an emergency, patients followed the usual procedure of going to the emergency department of primary care centers or hospitals.

Patients in both groups received the same routine care and did not undergo any interventions, whether diagnostic or monitoring, other than those planned according to routine clinical practice. Clinical data and antidiabetic treatment details were collected from the clinical history of patients and from information provided by the patient during the study visit and entered into the electronic case report form. Laboratory parameters, including HbA_1c_, low-density lipoprotein (LDL) cholesterol, and high-density lipoprotein (HDL) cholesterol, were taken from blood samples of all patients collected at baseline and thereafter, following local clinical practice until study completion or early study discontinuation.

The main evaluations compared groups 1 and 2 at week 24. The primary outcome of the study was to evaluate whether empowerment would reduce the proportion of patients persisting without metabolic control at week 24. The primary study glycemic target was an HbA_1c_ level ≤7.5% with a reduction in HbA_1c_ of ≥0.5% at week 24. Other secondary predefined study glycemic targets were HbA_1c_≤8%, HbA_1c_≤7%, and individualized HbA_1c_ targets for each patient at week 24, as established by the investigators. The absolute HbA_1c_ change at week 24 versus baseline was also a predefined secondary end point. In addition, mean changes in the body weight, BMI, blood pressure, LDL and HDL cholesterol levels, physical activity (measured as metabolic equivalent of task in min/week), and patient adherence to treatment were measured. Patient satisfaction with the DeMpower app and experience with health care received were also assessed. Finally, the mean number of symptomatic and asymptomatic hypoglycemic events (≤70 mg/dL) registered at emergency departments from baseline to week 24 between groups 1 and 2 was determined.

Questionnaires were used to evaluate study outcomes related to the degree of physical activity (International Physical Activity Questionnaire [IPAQ]), patient adherence to treatment (Medication Adherence Report Scale [MARS-5]), satisfaction with the DeMpower app (Diabetes Treatment Satisfaction Questionnaire status [DTSQs] version), and experience with health care received (Instrumento de Evaluación de la eXperiencia del PAciente Crónico [IEXPAC]) [19,34-37]. In this study, the short-form IPAQ was used, consisting of 4 generic domains with 7 questions in total for use in either interviews or self-administered methods [34]. The MARS-5 is a 5-item scale that includes questions about the way patients take their medicines and whether they forget to take them. Patients report agreement with statements about medicines using a 5-point Likert scale (from “always” [scored as 1] to “never” [scored as 5]). The maximum total score for all questions answered as “never” is 25 [35]. The DTSQs is an 8-item questionnaire, with 6 questions assessing treatment satisfaction and the other 2 assessing the perceived frequency of hyperglycemia and hypoglycemia. Each item is scored from 6 (ie, very satisfied) to 0 (ie, very dissatisfied), with the treatment satisfaction scale ranging from 36 (ie, very satisfied) to 0 (ie, very dissatisfied) and the perceived frequency of hyperglycemia and hypoglycemia scores ranging from 6 (ie, most of the time) to 0 (ie, none of the time) [36]. The IEXPAC is a 12-item scale that includes 11 questions plus 1 more conditional question about the experience of patients with chronic conditions regarding the health care and social attention that they have received. Items are answered as never (0 points), seldom (2.5 points), sometimes (5 points), most times (7.5 points), and always (10 points). The overall score of the 11 questions is calculated as their average score and ranges from 0 to 10. The additional question (item 12) is reported separately and ranges from 0 to 10 [19,37].

Assuming a bilateral contrast, an alpha risk of .05, a power of 80%, a proportion of response of 50% for each group and a patient loss of ≤13%, 100 patients were needed in group 1 (DeMpower app–empowered patients) and 50 patients in group 2 (control group) to detect a difference equal to or higher than 25% between both groups with regard to the primary study objective. For the descriptive analysis, quantitative variables were described with measures of centralization and dispersion (mean and SD), whereas qualitative variables were described by their absolute (N) and relative (%) frequencies. To compare 2 means between groups, parametric (Student *t* test) and nonparametric (Mann-Whitney *U* test) tests were used, as required. Categorical variables were compared with the chi-square or the Fisher exact test, when appropriate. Hypothesis tests were 2-tailed in all cases, with a significance level of .05. The evolution of HbA_1c_ throughout treatment was evaluated using a general linear model of repeated measures. Absences of data were not accounted for and were considered missing data. Statistical analyses were performed using SPSS (version 22.0 or higher; IBM Corp).

### Ethics Approval

The study was approved by the following ethics committees: Institut Universitari d’Investigació en Atenció Primària Jordi Gol (reference 5OB18/010), General University Hospital of Elda, Central Research Commission of Madrid, Murcian Health Service, and Health Areas of León and Bierzo.

## Results

**Figure 1:**
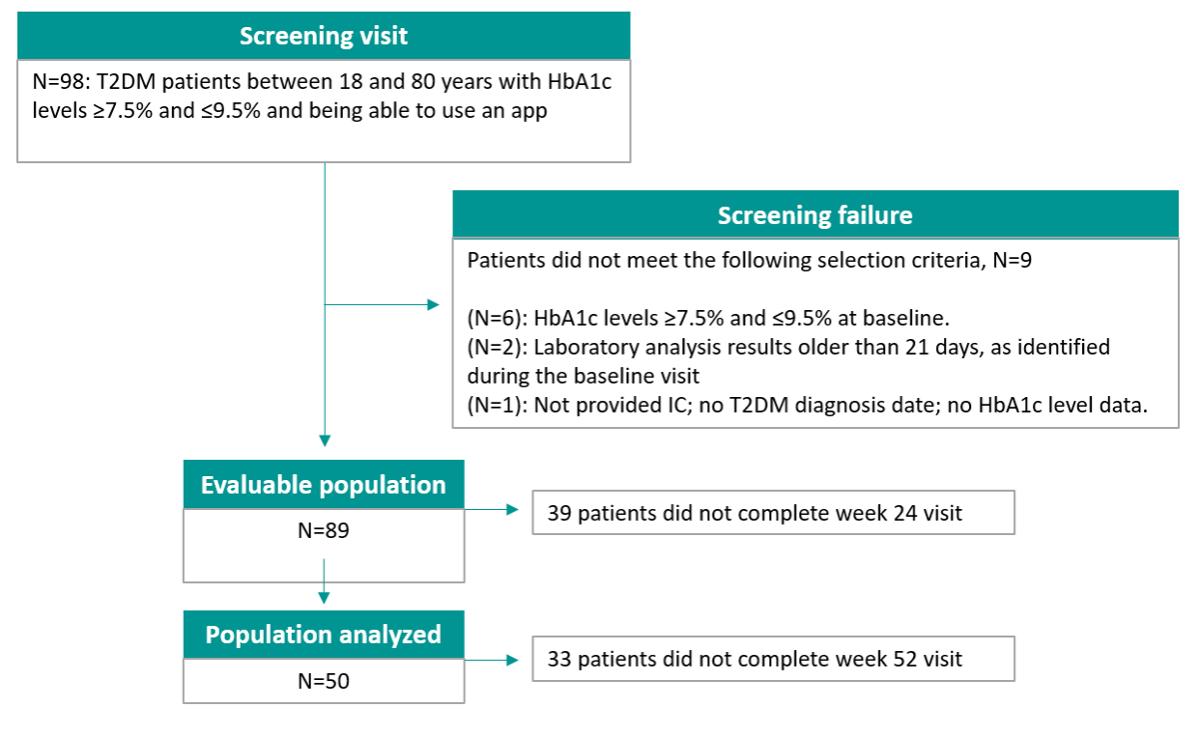
Study flowchart. HbA_1c_: hemoglobin A_1c_; IC: informed consent; T2DM: type 2 diabetes mellitus.

Due to the COVID-19 pandemic, the study was stopped prematurely (July 2020), with a relevant impact on both the recruitment and follow-up of patients. In addition, the primary hypothesis of the study, which was based on the empowerment of patients using the DeMpower app, could have been affected by the generally altered lifestyles of the patients during and after the COVID-19 lockdown period, both in the empowered and control groups. At the time of study discontinuation, 98 patients had been recruited in 15 of the 25 participating sites across Spain. Among these, 9 patients were excluded, as they did not meet the selection criteria and 89 were evaluable. Many of the patients were not able to attend visits and procedures due to the lockdown, and finally, 50 patients (33 patients in group 1 and 17 patients in group 2) completed the study visit at week 24 and were considered valid for the final analysis of the main study end points. At week 52, the number of patients remaining in groups 1a, 1b, and 2 were 6, 5, and 6, respectively (Figure 1). No patients abandoned the study due to an inability to adapt to the DeMpower app.

The baseline clinical characteristics of the study population are shown in Table 1. The groups were well balanced, without statistically significant between-group differences regarding clinical characteristics or baseline treatments, except for the presence of transient ischemic attack (no patients in group 1 vs 3 patients in group 2, *P*=.04) and the use of glinides (0 patients in group 1 vs 3 patients in group 2, *P*=.03). The mean age of the patients was 64 (SD 8) years, with 25% (13/50) older than 69 years, and 50% (25/50) of all patients were aged between 59 and 69 years. Overall, 66% (33/50) were male and 96% (48/50) were Caucasian; the mean diabetes duration was 10 (SD 6) years, and the mean BMI was 29.7 (SD 4.9) kg/m^2^. Complications associated with diabetes were not observed in 70% (23/33) of the patients in group 1 and 71% (12/17) of the patients in group 2 (*P*>.99). The majority of patients had at least 1 comorbidity (70% vs 59%, respectively; *P*=.53), with cardiovascular disease being the most common (61% vs 47%, respectively; *P*=.39). At baseline, the mean (SD) HbA_1c_ values were 8.2 (0.5) and 8.3 (0.6), respectively (*P*=.57). The most commonly prescribed antidiabetic drugs were metformin (88% vs 100%, respectively; *P*=.29), dipeptidyl peptidase-4 inhibitors (61% vs 41%; *P*=.24), sodium-glucose cotransporter-2 inhibitors (42% vs 53%; *P*=.56), and sulphonylureas (46% vs 18%; *P*=.40).

**Figure 2:**
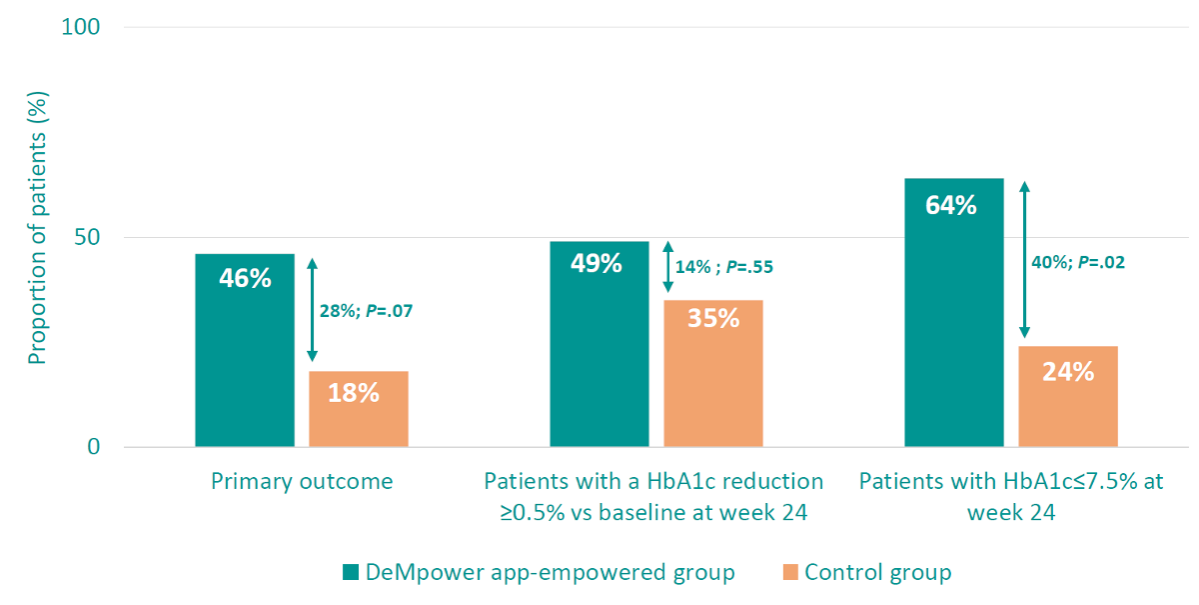
Primary composite outcome and individual components. Primary composite outcome refers to the proportion of patients achieving the study glycemic target (HbA_1c_≤7.5% with a reduction in HbA_1c_≥0.5% with respect to baseline value) at week 24. HbA_1c_: hemoglobin A1c.

Regarding the primary metabolic objective, there was a trend toward a higher proportion of people with T2DM achieving HbA_1c_ levels ≤7.5% with a reduction of ≥0.5% in HbA_1c_ with respect to the baseline value at week 24 (primary outcome) in group 1 compared with that in group 2 (46% vs 18%; *P*=.07), and this reached statistical significance when considering the proportion of people with T2DM achieving HbA_1c_ levels ≤7.5% at week 24 (64% vs 24%, respectively; *P*=.02; Figure 2).

**Figure 3:**
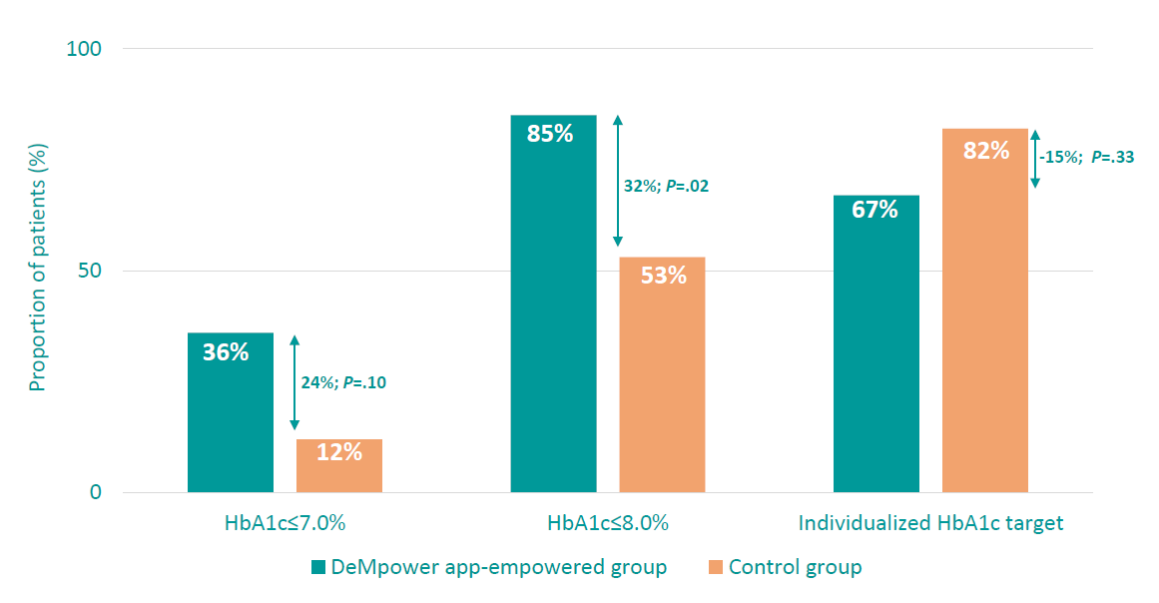
Proportion of patients with HbA_1c_≤7%, HbA_1c_≤8%, and individualized HbA_1c_ target established by the investigator at week 24. HbA_1c_: hemoglobin A_1c_.

When analyzing the percentage of patients with HbA_1c_ levels ≤7% at week 24 (36% vs 12%, respectively; *P*=.1) or the proportion of patients controlled according to the individualized HbA_1c_ objectives for each patient established by the investigators (67% vs 82%, respectively; *P*=.33), no significant between-group differences were observed. However, more patients in group 1 achieved significant HbA_1c_ levels ≤8% at week 24 (85% vs 53%, respectively; *P*=.02; Figure 3).

**Figure 4:**
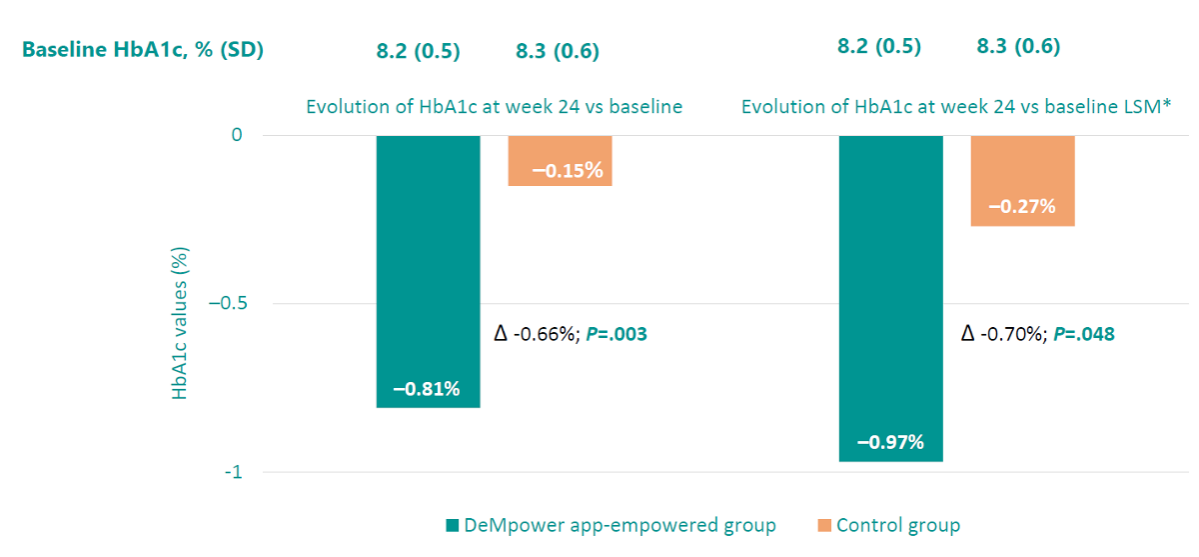
Changes in HbA_1c_ from baseline to week 24. HbA_1c_: hemoglobin A_1c_. *Least square means with adjustments for changes in antidiabetic treatment, age, sex, duration of diabetes, smoking status, socioeconomic status, educational level, and employment situation.

HbA_1c_ levels from baseline to week 24 significantly decreased to a higher extent in group 1 versus group 2 (−0.81 [0.89] vs −0.15 [1.03]; mean difference −0.66%; *P*=.03). This statistically significant difference remained after adjusting for changes in antidiabetic treatment, age, sex, duration of diabetes, smoking status, socioeconomic status, educational level, and employment situation (Figure 4).

No symptomatic and asymptomatic hypoglycemic events (≤70 mg/dL) from baseline to week 24 were reported in any of the groups at emergency departments both from primary care centers and hospitals. With regard to antidiabetic drugs, there were no statistically significant differences in treatment between the groups at week 24. In both groups, there was an overall increase in the prescription of antidiabetic agents at week 24 (Table S1 in Multimedia Appendix 1).

The evolution of the BMI, systolic blood pressure, diastolic blood pressure, LDL, HDL, and physical activity from the baseline visit to week 24 is shown in Table S2 in Multimedia Appendix 1. Although no statistically significant between-group differences were observed, there was a positive trend for group 1, with relevant reductions in the BMI and blood pressure, and an increase in physical activity.

The MARS-5 responses showed similar adherence to treatment in both groups at week 24, but with a positive trend in group 1. Patient satisfaction (DTSQs) and experience with the health care system (IEXPAC) were positive in both groups, with no significant between-group differences (Table S3 in Multimedia Appendix 1).

Due to the small number of patients completing the week 52 visit (17/50, 34%), no analysis was performed for the study exploratory end points at this time.

**Table 1:** Baseline sociodemographic characteristics of patients completing 24 weeks of follow-up.

Characteristic		Group 1 (n=33)	Group 2 (n=17)	*P* value
Age (years), mean (SD)		63.3 (6.4)	64.4 (9.5)	.46
Sex (male), n (%)		22 (66.7)	11 (64.7)	>.99
**Race, n (%)**				>.99
	Caucasian	32 (97)	16 (94.1)	
	Other	1 (3.0)	1 (5.9)	
**Educational level, n (%)**				.32
	Primary	10 (30.3)	8 (47.1)	
	Secondary	14 (42.4)	3 (17.6)	
	Higher education	8 (24.2)	5 (29.4)	
	Unknown	1 (3.0)	1 (5.9)	
**Professional situation, n (%)**				.94
	Unemployed	2 (6.1)	1 (5.9)	
	Employee	9 (27.3)	4 (23.5)	
	Autonomous	3 (9.1)	2 (11.8)	
	Retired	17 (51.5)	8 (47.1)	
	Other	2 (6.1)	2 (11.8)	
**Lifestyle habits, n (%)**				.06
	Active smoker	8 (24.2)	0 (0.0)	
	Ex-smoker	15 (45.5)	8 (47.1)	
	Never been a smoker	10 (30.3)	9 (52.9)	
	Unknown	0 (0.0)	0 (0.0)	
Time from T2DM^a^ diagnosis to study inclusion (years), mean (SD)		10.3 (7.3)	10.6 (4.6)	.47
**Complications associated with T2DM disease, n (%)**				
	None	23 (69.7)	12 (70.6)	>.99
	Microalbuminuria	6 (18.2)	1 (5.9)	.40
	Peripheral vascular disease	3 (9.1)	1 (5.9)	>.99
	Ischemic heart disease	2 (6.1)	1 (5.9)	>.99
	Neuropathy	2 (6.1)	2 (11.8)	.60
	Stroke	1 (3.0)	3 (17.6)	.11
	Retinopathy	1 (3.0)	1 (5.9)	>.99
	Transient ischemic attack	0 (0.0)	3 (17.6)	*.04* ^b^
	Heart failure	1 (3.0)	0 (0.0)	>.99
	Other complications	2 (6.1)	1 (5.9)	>.99
≥**1 comorbidity, n (%)**		23 (69.7)	10 (58.8)	.53
	Cardiovascular disease	20 (60.6)	8 (47.1)	.39
	Musculoskeletal disorder	10 (30.3)	5 (29.4)	>.99
	Endocrine disorder	7 (21.2)	3 (17.6)	>.99
	Neurological/psychiatric disorder	5 (15.2)	4 (23.5)	.47
	Gastrointestinal disorder	3 (9.1)	2 (11.8)	>.99
	Respiratory disease	5 (15.2)	2 (11.8)	>.99
	Hematological disease	4 (12.1)	0 (0.0)	.29
	Renal disease	2 (6.1)	1 (5.9)	>.99
	Infectious disease	2 (6.1)	1 (5.9)	>.99
	Cancer	2 (6.1)	2 (11.8)	.60
	Autoimmune disease	1 (3.0)	0 (0.0)	>.99
**Physical examination, mean (SD)**				
	BMI (Kg/m^2^)	30.2 (5.3)	28.7 (4.1)	.40
	SBP^c^ (mmHg)	137 (16.6)	130 (15.8)	.26
	DBP^d^ (mmHg)	79.5 (9.5)	76.1 (7.5)	.08
**Laboratory parameters, mean (SD)**				
	HbA_1c_^e^ (mg/dL)	8.2 (0.5)	8.3 (0.6)	.57
	Glucose	173.0 (35.3)	171.0 (38.9)	.84
	HDL^f^ cholesterol (mg/dL)	47.6 (18.1)	47.4 (11.2)	.58
	LDL^g^ cholesterol (mg/dL)	96.9 (29.7)	98.5 (34.6)	.85
	Individualized HbA_1c_ target	7.0 (0.2)	7.2 (0.3)	.05
	HbA_1c_ (mg/dL), value closest to week 24	7.9 (0.9)	8.2 (1.0)	.56
**Antidiabetic drugs^h^, n (%)**				
	Metformin	29 (87.9)	16 (94.1)	.49
	DPP-4^i^ inhibitors	19 (57.6)	8 (47.1)	.48
	SGLT2^j^ inhibitors	11 (33.3)	8 (47.1)	.34
	Sulfonylurea	14 (42.4)	3 (17.6)	.08
	GLP1^k^ receptor agonists	3 (9.1)	1 (5.9)	.69
	Glinides	0 (0.0)	3 (17.6)	*.03*
	Glitazones	1 (3)	0 (0.0)	.98

^a^T2DM: type 2 diabetes mellitus.

^b^Italics indicate significant *P* values <.05.

^c^SBP: systolic blood pressure.

^d^DBP: diastolic blood pressure.

^e^HbA_1c_: hemoglobin A_1c_.

^f^HDL: high-density lipoprotein.

^g^LDL: Low-density lipoprotein.

^h^Patients may have been indicated as receiving more than one antidiabetic drug.

^i^DPP-4: dipeptidyl peptidase-4.

^j^SGLT2: sodium-glucose cotransporter-2.

^k^GLP1: glucagon-like peptide-1.

## Discussion

The results from this study suggest that patient empowerment using the DeMpower app might improve metabolic control in people with T2DM who do not achieve HbA_1c_ targets with the standard care, possibly leading to a more efficient management of the disease.

The COVID-19 pandemic lockdown had a direct impact on the recruitment and follow-up of patients, reducing the planned study size. In addition, during lockdown, patients were not able to practice outdoor physical activities; some patients might have had uncontrolled dietary habits and physical access to health care providers was limited, leading to impaired metabolic control in both groups. Additionally, this could have also impacted patient-reported outcomes (ie, physical activity, adherence, as well as satisfaction and experience questionnaires). In fact, many research projects unrelated to COVID-19 have been substantialy reduced or even suspended due to legal restrictions or logistical, staffing, or operational concerns worldwide, as well as because of lockdowns or restrictions. Thus, a more flexible approach that ensures participant safety is warranted during the COVID-19 pandemic, under the good clinical practice umbrella [38]. In this context, investigating the impact of telemonitoring and telemedicine in patients with chronic conditions such as T2DM should be considered a priority, as it may facilitate better disease control [39,40].

The study groups were well balanced. The majority of patients were aged >60 years, had at least 1 comorbidity, and were taking more than 1 antidiabetic drug. This is in line with the clinical profiles reported in other studies of people with T2DM [10,41,42], indicating that patients included in our study were likely to be representative of the Spanish population with T2DM.

In our study, there was a trend in the primary outcome with a higher numerical proportion of empowered patients achieving the study glycemic target at week 24 compared to those in the control group. The between-group differences were statistically significant for secondary outcomes such as HbA_1c_ levels ≤7.5% and ≤8% at week 24, as well as absolute HbA_1c_ reduction at week 24, even after adjusting for several clinical characteristics including treatment modification. In particular, the 0.66% difference in HbA_1c_ levels between groups is clinically relevant and was achieved without increasing the risk of hypoglycemia. This is particularly remarkable given that there were no differences regarding the use or modification of antidiabetic drugs between both groups. These results support the clinical utility of the DeMpower app as a home digital patient empowerment and communication tool that might help patients achieve glycemic control that is independent of the antidiabetic treatment. Similarly, previous studies have also shown the benefits of home-based digital patient empowerment tools in the control of T2DM [23-33,43-47]. For example, the ValCrónic study [30] showed that the proportion of people with HbA_1c_≥8% decreased significantly (by 44%) after 1 year of telemonitoring. In our study, 85% of patients using the digital tool achieved HbA_1c_≤8%, compared to 53% in the control group (absolute difference 32%; relative difference 60%; *P*=.02). Additionally, meta-analyses of randomized controlled trials on telemedicine interventions have confirmed significant improvements in the management of diabetes compared with standard care [44,45]. The use of home digital tools for people with T2DM empowerment and metabolic control has become even more important during the COVID-19 pandemic, as during this period, metabolic control among people with T2DM has worsened [13-16]. In contrast, glycemic values in people with type 1 diabetes significantly improved during the COVID-19 lockdown, which may be associated with positive changes in self-care and digital diabetes management [16]. This reinforces the importance of improving self-care management using digital tools in T2DM and is in line with the DeMpower study results, suggesting that eHealth and telemedicine could reduce the negative impact of the COVID-19 pandemic and might be relevant in the digital health framework.

People with T2DM often present other comorbidities such as hypertension, dyslipidemia, obesity, and renal or cardiovascular disease [41,42]. Consequently, to reduce the cardiovascular burden in T2DM, it is necessary to implement a comprehensive approach that includes not only glycemic control but also blood pressure, lipid profile, body weight, and physical activity [48]. In our study, there was a positive trend for some of these variables in patients who used the DeMpower app. Additionally, considering that the lockdown during COVID-19 had a negative impact on metabolic and weight control [13-16], it is likely that with a larger sample size, these differences would have reached statistical significance. Besides, a recent meta-analysis of 43 studies reported a positive impact of telemedicine not only on HbA_1c_ but also on diastolic blood pressure, weight, and mental and physical quality of life, among people with T2DM [45]. Moreover, in the IDIATel randomized controlled trial [49] that compared telemedicine case management to routine care, greater reductions in LDL cholesterol and systolic and diastolic blood pressure levels were achieved with telemedicine.

Patient satisfaction with treatment is important to improve medication adherence [50]. Although our study did not show significant differences between groups, previous studies have shown an improvement with telemedicine [45]. Finally, the experience of patients regarding the health care attention received, evaluated with the IEXPAC tool, showed that there was opportunity for improvement for both groups, without significant differences. Similar results have been previously obtained regarding the information that patients receive or can access [19,37].

This study has some limitations. As noted earlier, the most relevant limitation is that the COVID-19 pandemic led to a premature study termination, and consequently, the estimated sample size of 150 patients could not be achieved. This might have impacted the statistical power for the assessment of the main study outcome. As this study was designed to collect information available in routine clinical practice at the participating sites, some data were unavailable, limiting the validity of the study results. Likewise, the appearance of bias derived from the unsuccessful use of digital tools could not be ruled out, but this was expected to be minimized by the selection of patients with a proven ability to use home mobile apps on their smartphones.

In summary, the DeMpower study results strongly suggest that patient empowerment through a home digital tool might lead to more effective metabolic control and consequently to more effective achievement of the clinical objectives in people with T2DM. This study reinforces the importance of using telemedicine and new technologies for patient empowerment and metabolic control, especially in the digital health scenario. Moreover, these findings appear to be crucial during situations with limited patient access to health care and negative health consequences, such as the COVID-19 pandemic.

## Appendix

Multimedia Appendix 1 Supplementary information.

## Abbreviations

DTSQs: Diabetes Treatment Satisfaction Questionnaire status
HbA_1c_: hemoglobin A_1c_
HDL: high-density lipoprotein
IEXPAC: Instrumento de Evaluación de la Experiencia del Paciente Crónico
IPAQ: International Physical Activity Questionnaire
LDL: low-density lipoprotein
MARS-5: Medication Adherence Report Scale
T2DM: type 2 diabetes mellitus
