# Supplementary material for: Effects of a Digital Patient Empowerment and Communication Tool on Metabolic Control in People With Type 2 Diabetes: The DeMpower Multicenter Ambispective Study
Source: JMIR Diabetes. 2022 Oct 3;7(4):e40377. doi: 10.2196/40377 (PMC9577714; doi:10.2196/40377)
Supplement: Multimedia Appendix 1 [file diabetes_v7i4e40377_app1.docx]

**Supplementary Figure 1. Study Design**


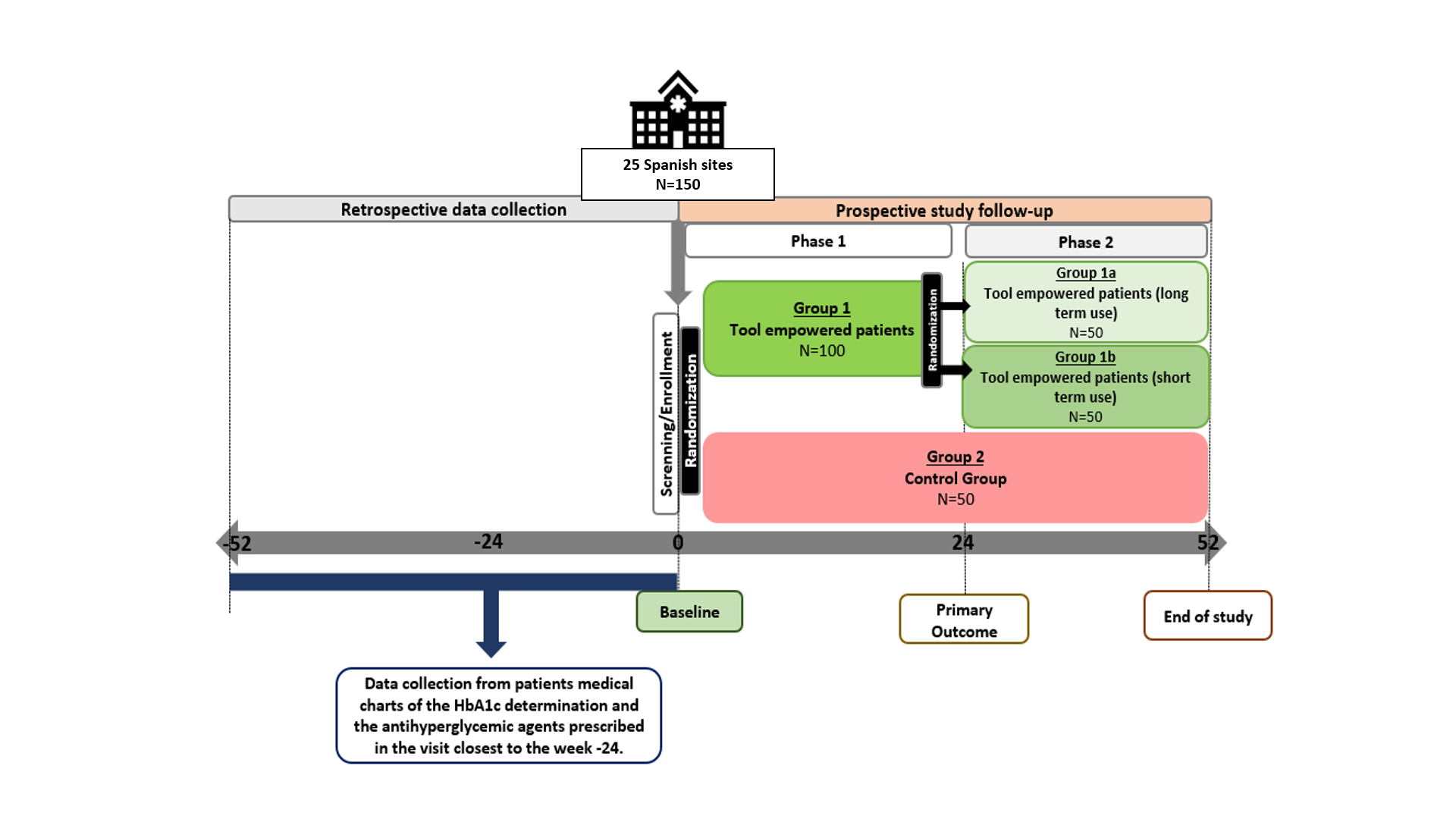


**Supplementary Figure 2. DeMpower App**


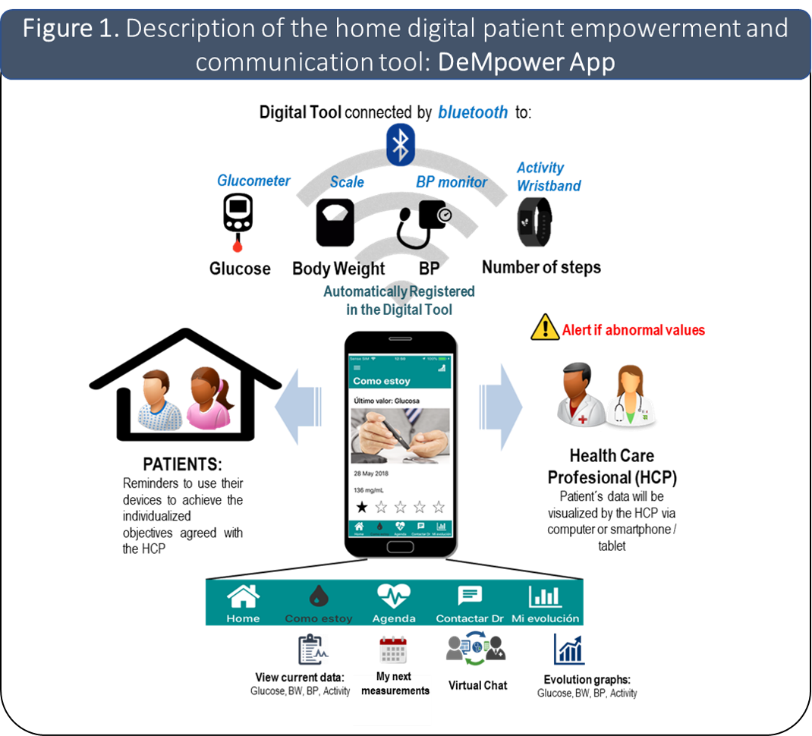


BP: blood pressure; BW: body weight.

**Supplementary Table 1: Changes in antidiabetic drugs used from baseline to week 24**

|  | **Group 1**  **(N=33)** | **Group 2**  **(N=17)** | **P** | **Group 1 (n=33)** | **Group 2 (n=17)** | **P** | **Group 1** | **Group 2** | **P** |
| --- | --- | --- | --- | --- | --- | --- | --- | --- | --- |
|  | **Baseline** | | | **Week 24** | | | **Difference week 24-baseline (∆)** | | |
| Metformin | 29 (87.9) | 17 (100) | 0.29 | 24 (72.7) | 17 (100) | 0.07 | 0 | 0 | - |
| DPP-4 inhibitors | 20 (60.6) | 7 (41.2) | 0.24 | 22 (66.7) | 9 (52.9) | 0.34 | 2 (6.1) | 2 (11.8) | 0.60 |
| SGLT2 inhibitors | 14 (42.4) | 9 (52.9) | 0.56 | 15 (45.5) | 8 (47.1) | 0.91 | 2 (6.1) | 0 | 0.54 |
| Sulfonylurea | 15 (45.5) | 3 (17.6) | 0.07 | 15 (45.5) | 4 (23.5) | 0.13 | 1 (3.0) | 1 (5.9) | >0.99 |
| GLP1 receptor agonists | 3 (9.1) | 3 (17.6) | 0.40 | 4 (12.1) | 3 (17.6) | 0.59 | 1 (3.0) | 0 | >0.99 |
| Glinides | 0 | 3 (17.6) | 0.03 | 1 (3.0) | 1 (5.9) | 0.63 | 1 (3.0) | 0 | >0.99 |
| Glitazones | 1 (3.0) | 0 | >0.99 | 1 (3.0) | 0 | 0.98 | 0 | 0 | - |

DPP4: dipeptidyl peptidase-4; GLP1: glucagon-like peptide-1; SGLT2: sodium/glucose cotransporter 2.

**Supplementary Table 2.** **Changes in body mass index, systolic blood pressure, diastolic blood pressure, LDL cholesterol, HDL cholesterol, and MET-minutes/week of total physical activity from baseline visit to week 24**

|  | **Group 1 (n=33)** | **Group 2 (n=17)** | **P** |
| --- | --- | --- | --- |
| BMI (kg/m^2^), mean (SD) | -0.4 (1.3) | 0.1 (1.2) | 0.25 |
| SBP (mmHg), mean (SD) | -9.0 (16.1) | -2.5 (10.7) | 0.19 |
| DBP (mmHg), mean (SD) | -2.7 (9.8) | 1.2 (9.0) | 0.12 |
| HDL cholesterol (mg/dL), mean (SD) | 1.8 (6.3) | 3.8 (6.8) | 0.40 |
| LDL cholesterol (mg/dL), mean (SD) | -5.0 (25.0) | -9.6 (31.2) | 0.57 |
| MET-minutes/week of total physical activity, mean (SD) | 1,106 (4,263) | 892 (2749) | 0.81 |

BMI: body mass index; DBP: diastolic blood pressure; HDL: high-density lipoprotein; LDL: Low-density lipoprotein; MET: metabolic equivalents of task; SBP: systolic blood pressure; SD: standard deviation.

**Supplementary Table 3. Comparison of patient adherence (MARS), satisfaction (DTSQs) and experience with the healthcare system (IEXPAC) from baseline to week 24**

|  | **Group 1 (n=33)** | **Group 2 (n=17)** | **P** |
| --- | --- | --- | --- |
| **Patient adherence** | | | |
| Total score MARS- questionnaire (baseline), mean (SD) | 23.6 (2.1) | 23.3 (1.5) | 0.08 |
| Total score MARS- questionnaire (week 24), mean (SD) | 24.2 (1.4) | 23.0 (2.7) | 0.05 |
| Variation in total score MARS- questionnaire, mean (SD) | 0.3 (1.7) | -0.06 (2.6) | 0.52 |
| **Patient satisfaction** | | | |
| **Total score of the DTSQ-questionnaire at baseline visit** | | | |
| Total score of DTSQ- questionnaire items 1, 4-8, mean (SD) | 29.5 (4.4) | 29.9 (5.0) | 0.66 |
| Total score of DTSQ- questionnaire item 2, mean (SD) | 3.5 (1.9) | 3.1 (1.8) | 0.23 |
| Total score of DTSQ- questionnaire item 3, mean (SD) | 0.8 (1.4) | 1.1 (1.4) | 0.20 |
| **Total score of the DTSQ-questionnaire at week 24** | | | |
| Total score of DTSQ- questionnaire items 1, 4-8, mean (SD) | 32.4 (3.6) | 32.6 (4.0) | 0.81 |
| Total score of DTSQ- questionnaire item 2, mean (SD) | 2.8 (1.6) | 2.5 (2.2) | 0.79 |
| Total score of DTSQ- questionnaire item 3, mean (SD) | 0.7 (1.2) | 0.7 (1.2) | 0.83 |
| **Variation in the DTSQ-questionnaire from baseline visit to week 24** | | | |
| Total score of DTSQ- questionnaire items 1, 4-8, mean (SD) | 1.8 (4.2) | 2.3 (4.4) | 0.94 |
| Total score of DTSQ- questionnaire item 2, mean (SD) | -0.6 (2.1) | -1.2 (2.5) | 0.83 |
| Total score of DTSQ- questionnaire item 3, mean (SD) | -0.5 (2.1) | -0.4 (1.6) | 0.95 |
| **Patient experience with the healthcare system** | | | |
| **Total score of the IEXPAC-questionnaire at baseline visit** | | | |
| Total score of IEXPAC- questionnaire items 1-11, mean (SD) | 7.6 (1.7) | 6.65 (1.9) | 0.08 |
| Total score of IEXPAC - questionnaire item 12, mean (SD) | 3.8 (4.6) | 5.28 (4.8) | 0.32 |
| **Total score of the IEXPAC-questionnaire at week 24** | | | |
| Total score of IEXPAC- questionnaire items 1-11, mean (SD) | 8.0 (1.2) | 7.53 (2.2) | 0.89 |
| Total score of IEXPAC - questionnaire item 12, mean (SD) | 3.6 (4.7) | 5.00 (5.8) | 0.68 |
| **Variation in the IEXPAC-questionnaire from baseline visit to week 24** | | | |
| Total score of IEXPAC- questionnaire items 1-11, mean (SD) | 0.1 (1.7) | 1.15 (3.1) | 0.20 |
| Total score of IEXPAC - questionnaire item 12, mean (SD) | 0.5 (4.7) | 0.0 (10.0) | 0.94 |

SD: standard deviation.
